# Allergic Reaction to Tropicamide Eye Drops: A Case Report

**DOI:** 10.7759/cureus.57945

**Published:** 2024-04-10

**Authors:** Seema Yelne, Meghana Pendam

**Affiliations:** 1 Nursing, Nursing tutor, Shalinitai Meghe College of Nursing, Datta Meghe Institute of Medical Sciences, Sawangi, Meghe, Wardha, Maharashtra, India., Wardha, IND; 2 Dermatology, Jawaharlal Nehru Medical College, Datta Meghe Institute of Higher Education and Research, Wardha, IND

**Keywords:** dermoscopic examination, periocular swelling, ophthalmic examination, eye drops, tropicamide, allergic reaction

## Abstract

This case report presents the clinical scenario of a 50-year-old man who developed swelling and itching around both eyes after applying tropicamide eye drops for an ophthalmic examination. The swelling appeared suddenly, progressed over time, and was accompanied by redness, watery discharge, and conjunctival congestion. A dermoscopic examination revealed congestion and erythema in the affected area. Visual acuity was compromised in the left eye. Prompt identification of the eyedrops as plain tropicamide with chlorbutol as a preservative enabled timely treatment with intravenous hydrocortisone and topical steroids, resulting in symptom improvement within two days. Allergic reactions to mydriatic agents such as tropicamide are infrequent but should be considered in patients with acute ocular symptoms post-application. This case underscores the importance of recognising and managing allergic reactions to ophthalmic medications for optimal patient care.

## Introduction

Allergic reactions to ophthalmic medications, although infrequent, can result in significant morbidity and may present with a wide range of clinical manifestations. Tropicamide, a commonly used mydriatic agent, has rarely been associated with allergic reactions [1]. Allergic reactions to ophthalmic medications can manifest as local or systemic symptoms, ranging from mild irritation to severe inflammation. These reactions may occur due to hypersensitivity to the active ingredients, preservatives, or other components of the eye drops [2]. While allergic conjunctivitis is well-recognized, allergic reactions to mydriatic agents like tropicamide are less common. Still, they can present with similar ocular symptoms, including redness, swelling, itching, and watery discharge [3].

Tropicamide is a synthetic anticholinergic agent commonly used to induce mydriasis and cycloplegia in ophthalmic examinations. It blocks the muscarinic acetylcholine receptors in the iris sphincter muscle and ciliary body, leading to pupil dilation and paralysis of accommodation [4]. While generally considered safe and effective, tropicamide has various adverse effects, including transient stinging or burning sensation upon instillation, conjunctival hyperemia, and blurred vision [5]. Although allergic reactions to tropicamide eye drops are rare, they have been reported in the literature. These reactions typically present as acute-onset swelling, erythema, and pruritus around the eyes, often accompanied by conjunctival congestion and discharge [6]. Systemic absorption of tropicamide can occur through the conjunctiva, nasal mucosa, or nasolacrimal duct, potentially leading to systemic toxicity symptoms such as tachycardia, dry mouth, urinary retention, and central nervous system effects [7].

Recognition of allergic reactions to tropicamide eye drops is crucial for prompt management and prevention of complications. Treatment may involve discontinuation of the offending medication, symptomatic relief with antihistamines or topical steroids, and, in severe cases, systemic corticosteroids or other immunomodulatory agents [8]. Healthcare providers should be vigilant in monitoring patients for adverse reactions to ophthalmic medications and consider alternative agents in individuals with a history of allergy or intolerance.

## Case presentation

**Figure 1:**
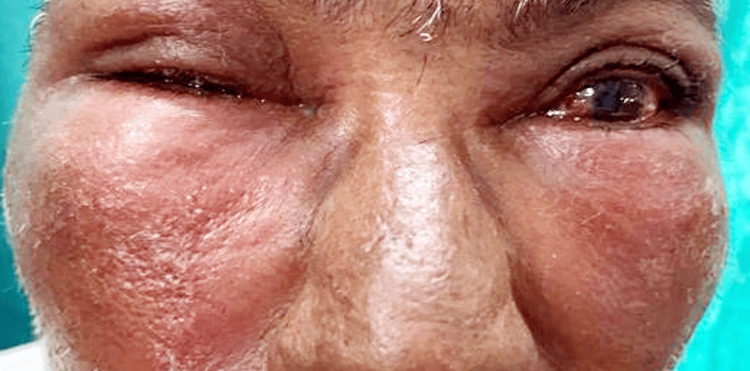
Swelling, lid edema, and watery discharge from the eyes

**Figure 2:**
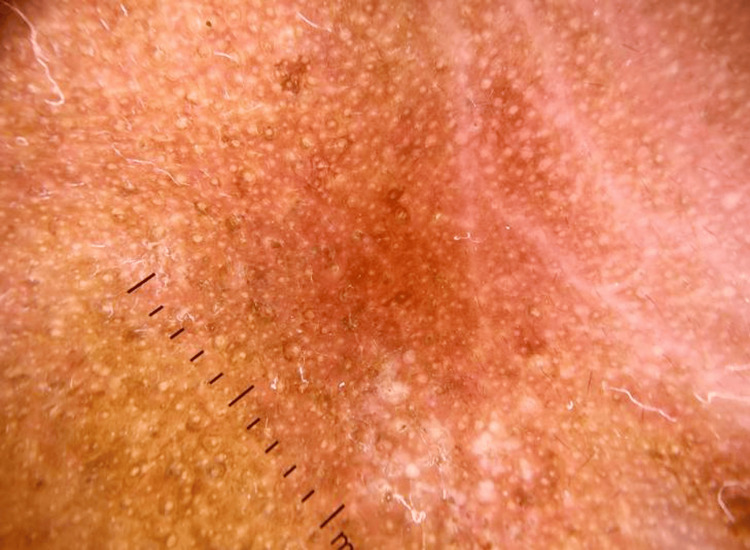
Dermoscopy of the affected site showing signs of inflammation

A 50-year-old man presented to the dermatology clinic with swelling and itching around both eyes for one day. He had applied eyedrops the day before for an ophthalmic examination. The swelling appeared suddenly, was painless, and progressed over time. He reported no fever, generalized swelling, or dizziness. Upon external examination, there was edema and redness around both eyes, extending from below the eyebrows to the cheekbones and up to the ears Figure 1. The skin over the swelling was red throughout. He also had swollen eyelids and watery discharge from both eyes. Dermoscopy of the affected area showed redness and congestion Figure 2. His visual acuity was 6/6 in the right eye and 6/60 in the left eye. Both eyes exhibited conjunctival congestion. The rest of the examination of both eyes' front and back portions was normal.

**Figure 3:**
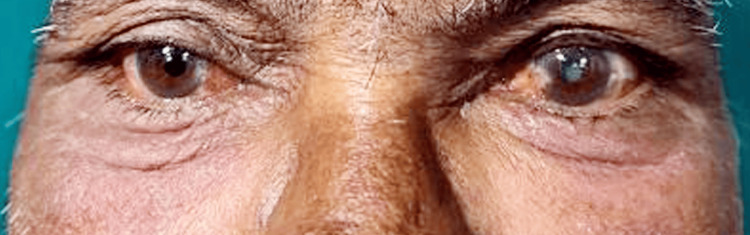
Subsided swelling and lid edema

Upon examination, the eyedrops were identified as plain tropicamide with chlorbutol as a preservative. He was treated with intravenous hydrocortisone and topical steroids, leading to improvement within two days as shown in Figure 3. Allergic reactions to mydriatic eyedrops are frequently overlooked. Tropicamide is a synthetic derivative of tropic acid and belongs to the parasympathomimetic group of drugs, similar to atropine. Systemic absorption can occur through the conjunctiva, nasal mucosa, or via the nasolacrimal duct into the gastrointestinal tract, potentially causing systemic toxicity symptoms.

## Discussion

Allergic reactions to ophthalmic medications are rare but can significantly affect patient care and clinical outcomes. In this case report, we presented a 50-year-old man who developed swelling, itching, and conjunctival congestion following the use of tropicamide eye drops for an ophthalmic examination. Prompt recognition and appropriate management led to the resolution of symptoms within two days. Tropicamide is a widely used mydriatic agent that blocks muscarinic acetylcholine receptors in the iris sphincter muscle and ciliary body, resulting in pupil dilation and paralysis of accommodation. While generally well-tolerated, tropicamide has been associated with various adverse effects, including ocular irritation, conjunctival hyperemia, and blurred vision [9]. Although rare, allergic reactions to tropicamide eye drops have been reported in the literature. As observed in our patient, these reactions typically present as acute-onset periocular swelling, erythema, pruritus, and conjunctival congestion [10].

The pathophysiology of allergic reactions to ophthalmic medications involves a type I hypersensitivity reaction mediated by immunoglobulin E (IgE) antibodies. Upon exposure to the allergen (in this case, tropicamide), mast cells and basophils release inflammatory mediators such as histamine, leukotrienes, and cytokines, leading to the characteristic signs and symptoms of allergic conjunctivitis [11]. Systemic absorption of tropicamide can exacerbate the allergic response, potentially leading to systemic symptoms such as tachycardia, dry mouth, and central nervous system effects [12]. Recognition of allergic reactions to ophthalmic medications is essential for prompt management and prevention of complications. In our case, the patient was promptly treated with intravenous hydrocortisone and topical steroids, leading to rapid improvement of symptoms. Discontinuation of the offending medication is crucial to prevent further exposure and recurrence of symptoms. In severe cases, systemic corticosteroids or other immunomodulatory agents may be required to control the allergic response [13].

Healthcare providers should be vigilant in monitoring patients for adverse reactions to ophthalmic medications, particularly those with a history of allergy or intolerance. Patient education regarding the potential side effects of prescribed medications is paramount to facilitate early recognition and management of adverse reactions. Additionally, alternative agents may be considered in individuals with a known history of allergy to specific ophthalmic medications.

## Conclusions

In conclusion, the case underscores the significance of recognizing allergic reactions to ophthalmic medications, particularly mydriatic agents like tropicamide, when patients present with ocular symptoms post-application. Although such reactions are rare, the manifestation of periocular swelling, redness, and conjunctival congestion, as seen in our patient, warrants prompt attention and appropriate management. Clinicians can expedite the resolution of symptoms and prevent potential complications by discontinuing the offending medication and administering symptomatic relief using topical or systemic steroids, as demonstrated in this case. Vigilance in monitoring patients for adverse drug reactions, especially in those with a history of allergy or intolerance, remains paramount. Furthermore, patient education regarding prescribed medications' potential side effects is crucial for early recognition and intervention. Looking forward, further research is needed to deepen our understanding of the mechanisms underlying allergic reactions to ophthalmic medications and to develop effective preventive and management strategies.
